# Impact of chronic kidney disease severity on causes of death after first-ever stroke: A population-based study using nationwide data linkage

**DOI:** 10.1371/journal.pone.0241891

**Published:** 2020-11-19

**Authors:** Hsin-Hsu Wu, Ting-Yu Chang, Chi-Hung Liu, Jr-Rung Lin, Chia-Wei Liou, Jiann-Der Lee, Tsung-I Peng, Meng Lee, Tsong-Hai Lee

**Affiliations:** 1 Kidney Research Center, Department of Nephrology, Chang Gung Memorial Hospital, Taoyuan, Taiwan; 2 Department of Medicine, Chang Gung University, Taoyuan, Taiwan; 3 Department of Neurology, Stroke Section, Chang Gung Memorial Hospital, Linkou Medical Center, Taoyuan, Taiwan; 4 Clinical Informatics and Medical Statistics Research Center, Chang Gung University, Taoyuan, Taiwan; 5 Department of Neurology, Stroke Section, Chang Gung Memorial Hospital, Kaohsiung Medical Center, Kaohsiung, Taiwan; 6 Department of Neurology, Chang Gung Memorial Hospital, Chiayi Branch, Chiayi, Taiwan; 7 Department of Neurology, Chang Gung Memorial Hospital, Keelung Branch, Keelung, Taiwan; Baker IDI Heart and Diabetes Institute, AUSTRALIA

## Abstract

**Background:**

Stroke is prevalent in patients with chronic kidney disease (CKD) and is associated with high mortality, but the causes of death after stroke among different CKD stages are not well known.

**Aims:**

We aimed to investigate whether the severity of CKD would impact on the causes of death after first-ever stroke.

**Methods:**

This retrospective multicenter cohort study included stoke patients with CKD between 2007 and 2012. The cause of death and date of death were ascertained by linking the National Death Registry Database of Taiwan. Clinical outcomes, 1-month, and 1-year mortality rates, and major causes of death were compared according to five CKD stages (G1 to G5) in the ischemic and hemorrhagic stroke separately.

**Results:**

Of these patients, 9,878 were first-ever ischemic stroke (IS) patients, and 1,387 were first-ever hemorrhagic stroke (HS) patients. Patients with CKD G5 had the highest one-year mortality rate with hazard ratio 5.28 [95%CI, 3.94–7.08] in IS and 3.03 [95%CI, 2.03–4.54] in HS when compared to G1 patients. Leading causes of one-year death after IS were stroke, cancer, and pneumonia in early (G1-3) CKD patients, while diabetes mellitus, CKD, and stroke itself contributed to the major mortality in CKD G5 patients. An inverse association between eGFR decrement and the proportion of deaths caused by stroke itself was observed in CKD G2-5 patients after IS. Stroke was the leading cause of one-year death among all CKD patients after HS.

**Conclusions:**

Asides from high mortality, late-stage CKD patients had different causes of death from early CKD patients after stroke. This study highlights the need to imply different treatment strategies in late-stage CKD post-stroke patients to improve their prognosis.

## Introduction

Stroke is the leading cause of mortality and disability worldwide [1, 2]. In the recent decade, chronic kidney disease (CKD) has been recognized as an important risk factor both for ischemic stroke (IS) and hemorrhagic stroke (HS) [3]. Stroke and CKD share common risk factors including old age, diabetes mellitus (DM), and hypertension [4]. CKD patients also have a high prevalence of non-traditional cardiovascular risk factors such as mineral and bone disorder, malnutrition, and inflammatory status. Thus, cerebrovascular diseases are common in CKD patients [5, 6].

While the mortality after stroke has declined over time due to better control of modifiable risk factors and better in-hospital management [7, 8], the death rate after stroke in CKD patients remains high, especially in those who progressed to late CKD [9, 10]. Late CKD patients not only suffered more stroke severity but also had higher mortality after stroke [11, 12]. However, there has been no study discussing the impact of different CKD stages on the causes of death in post-stroke patients. In the modern era of precision medicine, it is important to understand the causes of death after stroke in patients with different CKD severity, and have different treatment strategies according to the CKD stages. This study aims to investigate the major causes of death among post-stroke patients with varying stages of CKD. By linking two cohort databases: stroke registry data from the largest private health care system in Taiwan, and the National Death Registry Database of Taiwan, we assessed the relationship between the outcomes of stroke and the CKD stages.

## Methods

### Ethics approval and consent to participate

This study was approved by the Institutional Review Board of the Chang Gung Memorial Hospital, Taoyuan, Taiwan (IRB No: 201800379B1). Informed consent was waived because no identifiable information was used in this study.

### Data source and study population

This retrospective cohort study investigated all patients from 2007 to 2012 with a diagnosis of stroke registered in the Stroke Registry in Chang Gung Healthcare System (SRICHS), Taiwan. The Chang Gung Healthcare System in Taiwan has four hospitals, including two tertiary medical centers in Northern and Southern Taiwan and two local hospitals, which treated an average of 2.4 million people hospitalized per year. All patients with a primary diagnosis of stroke during emergency department visits or admissions were enrolled in this database. The registry database included stroke subtypes and special treatments for stroke. Detailed clinical information, including personal habits, medical history, laboratory data, medical imaging data, and clinical outcomes, was recorded and regularly validated by specifically trained people. The causes of death and date of death of the study cohort were ascertained by linking SRICHS with the National Death Registry Database of Taiwan. The underlying cause of death on the death certificate was coded according to the International Classification of Diseases, Tenth Revision, Clinical Modification (ICD-10-CM) in computerized data files. Detailed ICD-10-CM codes are listed in S1 Table.

### Exclusion criteria

Patients with missing data, who were undergoing dialysis, who received kidney transplantation, who received thrombolytic therapy or who had a non-primary hemorrhagic stroke were excluded.

### Glomerular filtration rate estimation and CKD classification

The estimated glomerular filtration rate (eGFR) of each study participant was calculated using the CKD Epidemiology Collaboration (CKD-EPI) equation [13]. This equation was found to be more accurate than the Modification of Diet in Renal Disease (MDRD) equation [14], and ethnic adjustment for Asian patients was not required [15]. Patients were grouped according to their eGFR: eGFR ≥ 90 ml/min/1.73 m^2^ staged as G1, eGFR 60–89 ml/min/1.73 m^2^ staged as G2, eGFR 44–59 ml/min/1.73 m^2^ staged as CKD G3, eGFR 15–29 ml/min/1.73 m^2^ staged as CKD G4, and eGFR < 15 ml/min/1.73 m^2^ staged as CKD G5.

### Statistical analysis

We used one-way ANOVA and chi-square tests for comparisons of numerical and categorical variables, respectively. Tukey’s honestly significant difference post hoc test was used if a statistically significant difference in group means was noted. Cox-proportional hazard regression was used to calculate the adjusted hazard ratio (HR) with the 95% confidence interval. Post-stroke survival analysis was done by using the Kaplan-Meier estimate. A significance threshold was defined if *p*<0.05. Patients with missing data were excluded from this study (estimated to be less than 1% of our stroke patients). Age, sex, hypertension, DM, atrial fibrillation, malignancy, and National Institute of Health Stroke Scale (NIHSS) score at onset were adjusted when estimating survival data and CKD stage-related post-stroke mortality rate HR. The analysis was performed using SAS (SAS Institute) version 9.4.

## Results

**Fig 1:**
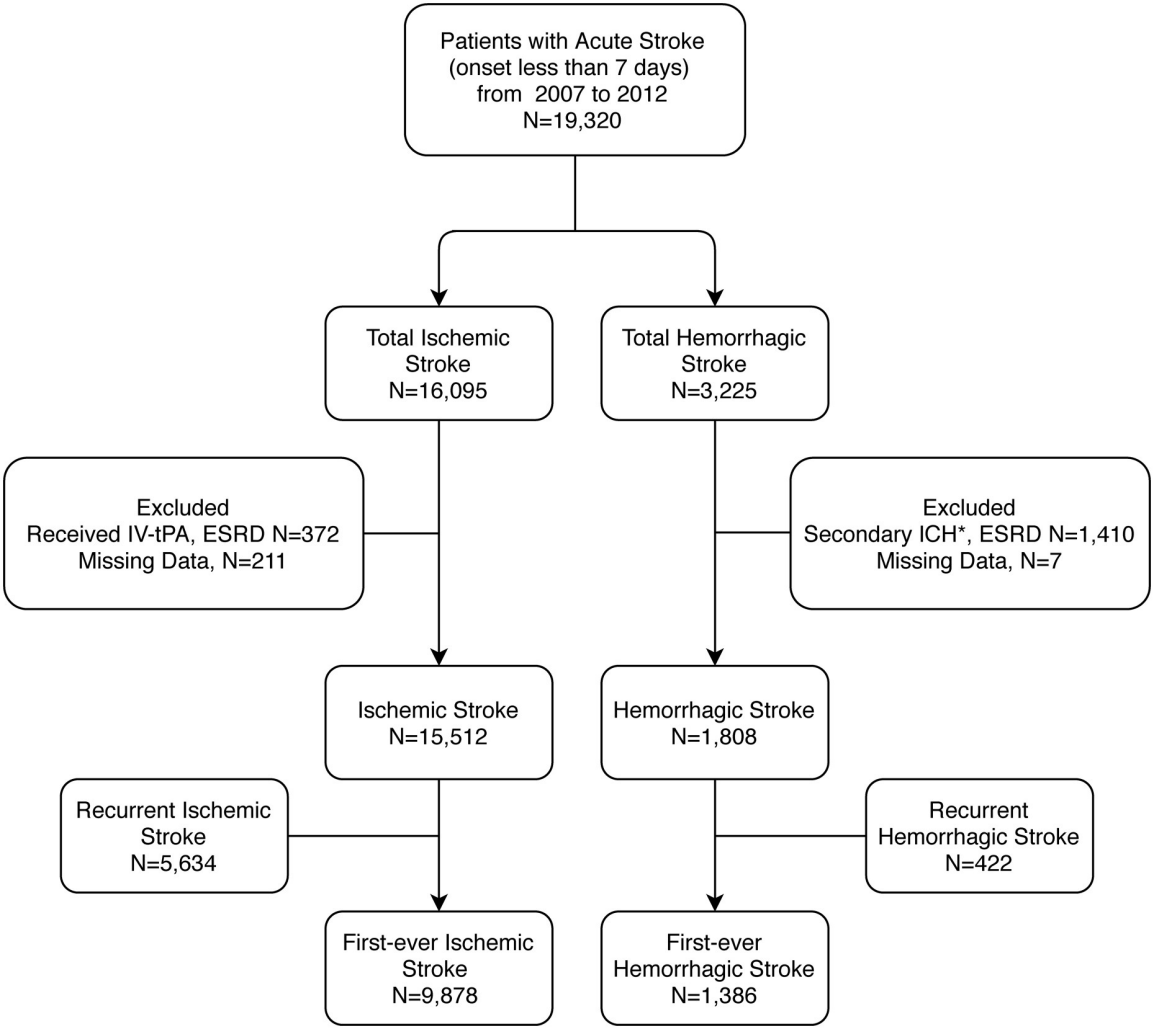
Flowchart of the study protocol. Flowchart of the study protocol. ESRD: end-stage renal disease; IV-rtPA: intravenous recombinant tissue plasminogen activator; ICH: intracerebral hemorrhage; *secondary ICH: traumatic brain injury, underlying vascular malformation, intracranial tumor, hemorrhagic conversion of an ischemic stroke.

From 2007 to 2012, a total of 19,320 acute stroke patients, including 16,095 IS patients and 3,225 HS patients, were identified in SRICHS. Patients with missing data, under dialysis, post kidney transplantation, post thrombolytic therapy, or non-primary HS were excluded. After exclusion, there were 15,512 IS patients and 1,808 HS patients. We further divided these patients into first-ever stroke and recurrent stroke. A total of 9,878 first-ever IS patients, and 1,386 first-ever HS patients were analyzed in this study. The study flow diagram is shown in Fig 1.

### Worse outcomes in late-stage CKD patients with first-ever stroke

Patients with first-ever IS were further divided into five groups (G1 to CKD G5) according to their eGFR. Detailed demographic data are shown in Table 1 and S2 Table. The NIHSS score, as an important parameter to quantify stroke severity and a strong predictor of outcome [16], was significantly higher in late-stage CKD patients (7.5±8.4 for CKD G5 versus 5.2±6.0 for G1, *p* <.0001). The Barthel Index and modified Rankin Scale (mRS) were evaluated for each stroke patient upon discharge for measuring the degree of disability and dependence. Higher degrees of disability upon discharge were noted in late CKD patients with higher mRS (2.7±1.8 for CKD G5 versus 2.0±1.6 for G1, *p* <.0001) and lower Barthel Index (63.4±37.5 for CKD G5 versus 77.6±32.2 for G1, *p* <.0001). Post-hoc analysis among different CKD stages was shown in S3 Table.

**Table 1:** Demographic data of first-ever stroke patients.

**First-ever ischemic stroke** eGFR (ml/min/1.73 m^2^)	**Total**	**G1** ≥90	**G2** 60–89	**CKD G3** 30–59	**CKD G4** 15–29	**CKD G5** <15	***p-*value**
Number	9,878	3,372	3,957	1,943	309	297	
Age, y/o	67.1±13.2	60.9±13.0	68.7±12.2	73.8±11.2	74.1±11.8	66.8±12.6	<.0001
Male, n (%)	5967 (60.4)	2095 (62.1)	2482 (62.7)	1089 (56)	156 (50.5)	145 (48.8)	<.0001
Admission NIHSS	5.9±7.0	5.2±6.0	5.6±6.8	6.8±7.8	7.8±8.8	7.5±8.4	<.0001
Hypertension, n (%)	7407 (75)	2247 (66.6)	2986 (75.5)	1639 (84.4)	272 (88.0)	263 (88.6)	<.0001
Atrial fibrillation, n (%)	1339 (13.6)	276 (8.2)	578 (14.6)	379 (19.5)	68 (22.0)	38 (12.8)	<.0001
Other heart diseases, n (%)	2744 (27.8)	624 (18.5)	1129 (28.5)	747 (38.4)	133 (43.0)	111 (37.4)	<.0001
Hyperlipidemia, n (%)	3168 (32.1)	1107 (32.8)	1221 (30.9)	647 (33.3)	111 (35.9)	82 (27.6)	0.0452
Diabetes mellitus, n (%)	3683 (37.3)	1155 (34.3)	1309 (33.1)	842 (43.3)	187 (60.5)	190 (64.0)	<.0001
Smoking, n (%)	3284 (33.2)	1295 (38.4)	1339 (33.8)	511 (26.3)	82 (26.5)	57 (19.2)	<.0001
Alcohol, n (%)	1669 (16.9)	741 (22.0)	649 (16.4)	227 (11.7)	25 (8.1)	27 (9.1)	<.0001
**Outcome**							
Mortality, n (%)							
1M	230 (2.3)	36 (1.1)	76 (1.9)	78 (4.0)	21 (6.8)	19 (6.4)	<.0001
12M	885 (9.0)	145 (4.3)	298 (7.5)	294 (15.1)	70 (22.7)	78 (26.3)	<.0001
Discharge BI	73.0±34.3	77.6±32.2	74.6±33.4	66.6±36.2	57.9±37.5	63.4±37.5	<.0001
Discharge mRS	2.2±1.7	2.0±1.6	2.2±1.7	2.5±1.7	2.9±1.7	2.7±1.8	<.0001
Length of stay (days)	10.9±12.5	10.1±13.8	10.5±10.8	12.1±12.5	13.5±13.9	14.0±15.4	<.0001
**First-ever hemorrhagic stroke** eGFR (ml/min/1.73 m^2^)	**Total**	**G1** ≥90	**G2** 60–89	**CKD G3** 30–59	**CKD G4** 15–29	**CKD G5** <15	***p-*value**
Number	1,386	560	491	204	43	88	
Age, y/o	60.5±14.3	56.8±13.5	61.9±14.4	66.3±14.2	63.3±14.3	60.7±13.2	<.0001
Male, n (%)	910 (65.7)	368 (65.7)	332 (67.6)	123 (60.3)	33 (76.7)	54 (61.4)	0.1646
Admission NIHSS	15.3±13.9	13.9±12.9	14.5±13.8	17.3±14.8	17.5±15.0	23.0±15.2	<.0001
Hypertension, n (%)	1155 (83.3)	433 (77.3)	422 (85.9)	180 (88.2)	40 (93)	80 (90.9)	<.0001
Atrial fibrillation, n (%)	69 (5.0)	18 (3.2)	26 (5.3)	19 (9.3)	1 (2.3)	5 (5.7)	0.0133
Other heart diseases, n (%)	158 (11.4)	44 (7.9)	66 (13.4)	35 (17.2)	4 (9.3)	9 (10.2)	0.003
Hyperlipidemia, n (%)	178 (12.8)	69 (12.3)	75 (15.3)	23 (11.3)	5 (11.6)	6 (6.8)	0.1926
Diabetes mellitus, n (%)	292 (21.1)	111 (19.8)	82 (16.7)	44 (21.6)	19 (44.2)	36 (40.9)	<.0001
Smoking, n (%)	559 (40.3)	236 (42.1)	193 (39.3)	78 (38.2)	19 (44.2)	33 (37.5)	0.7526
Alcohol, n (%)	484 (34.9)	234 (41.8)	167 (34.0)	55 (27.0)	13 (30.2)	15 (17.0)	<.0001
**Outcome**							
Mortality							
1M	211 (15.2)	61 (10.9)	63 (12.8)	44 (21.6)	7 (16.3)	36 (40.9)	<.0001
12M	319 (23.0)	90 (16.1)	100 (20.4)	66 (32.4)	12 (27.9)	51 (58.0)	<.0001
Discharge BI	44.2±40.4	47.8±39.9	47±40.8	37.7±40.2	36.4±36.3	24.5±36.7	<.0001
Discharge mRS	3.4±1.8	3.2±1.7	3.2±1.8	3.7±1.8	3.7±1.6	4.2±1.7	<.0001
Length of stay (days)	16.2±16.3	16±16.7	15.7±15.1	16.4±15.6	21.2±20.5	16.8±18.9	0.3057

n (%) = number (%); M±SD = mean± standard deviation; BMI: body mass index; NIHSS: National Institute of Health Stroke Scale; eGFR: estimated glomerular filtration rate; BI: Barthel index; mRS: modified Ranking Scale

First-ever HS patients were also divided into five subgroups according to their eGFR, as shown in Table 1. The NIHSS score was significantly higher in the CKD G5 group than in the normal eGFR groups (23.0±15.2 for CKD G5 versus 13.9±12.9 for G1: 13.9±12.9, *p* <.0001). HS patients with late-stage CKD maintained higher degrees of disability (mRS was 4.2±1.7 for CKD G5 patients versus 3.2±1.7 for G1 patients, *p* <.0001) and were more dependent (Barthel Index was 24.5±36.7 for CKD G5 patients versus 47.8±39.9 for G1, *p* <.0001) upon discharge (Table 1). Post-hoc analysis of these outcomes was shown in S4 Table.

### Higher mortality in late-stage CKD patients after first-ever stroke

The mortality rate of first-ever IS patients was higher among late-stage CKD patients than among early-stage CKD patients. This phenomenon was observed for both one-month and one-year mortality. One-month mortality rates were 1.1% for G1 patients and 6.4% for CKD G5. The one-year mortality rate increased with CKD stages, progressing from 4.3% in G1 patients up to 26.3% in CKD G5 patients (Table 1).

A similar trend of increasing mortality with decreasing eGFR was also noted in the HS group. The one-month mortality rate was 10.9% for G1 patients and increased markedly to 40.9% for CKD G5 patients. The one-year mortality rate was lowest in G1 patients (16.1%) and highest in CKD G5 patients (58%). More than half of CKD G5 patients died within one year after HS in our cohort (Table 1).

**Fig 2:**
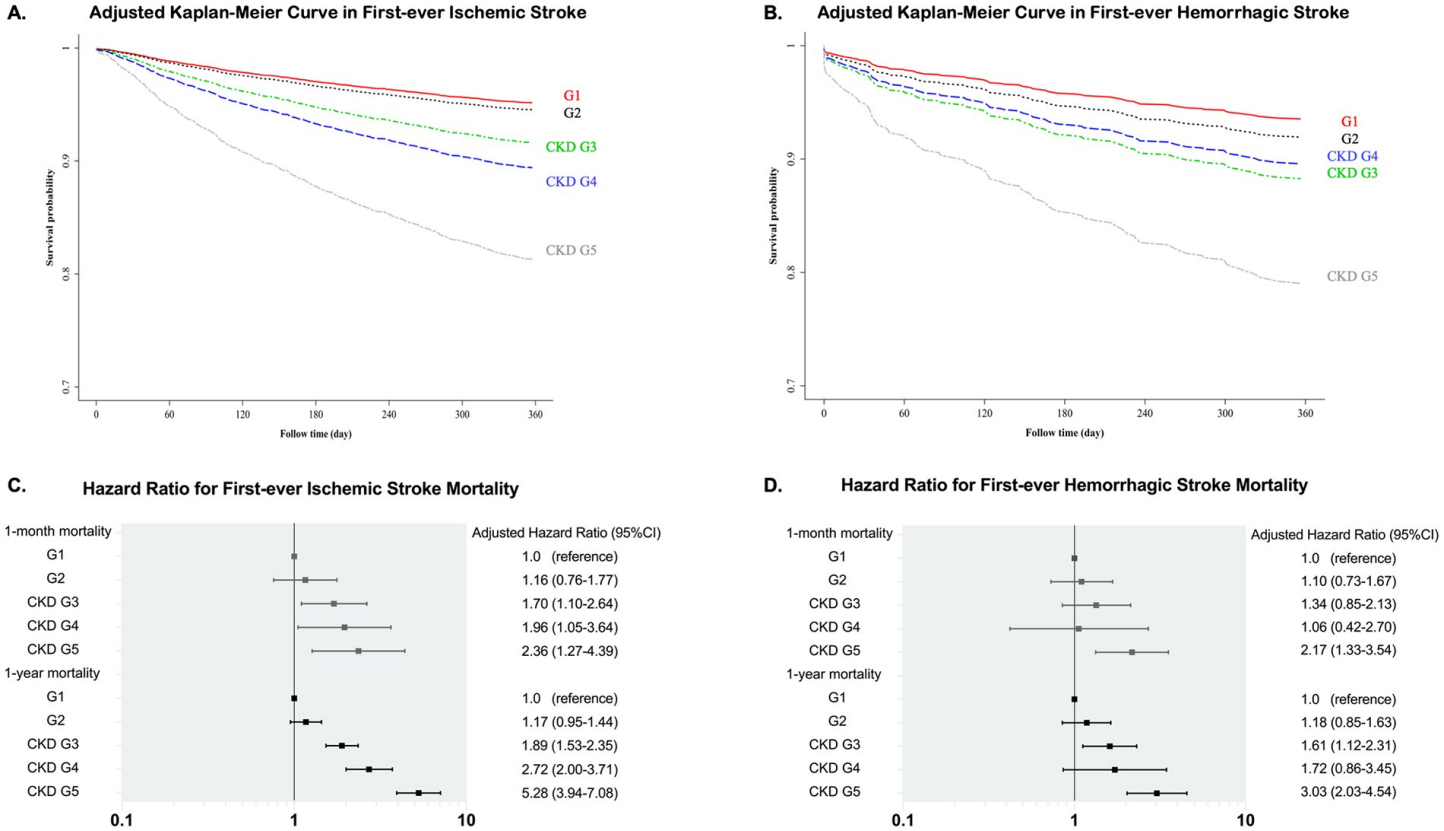
Survival curve of first-ever stroke patients and hazard ratio of post-stroke mortality among different CKD stages. (A) Adjusted Kaplan-Meier survival curve of first-ever ischemic stroke patients; (B) Adjusted Kaplan-Meier survival curve of first-ever hemorrhagic stroke patients. Adjusted factors include sex, age, hypertension, diabetes mellitus, atrial fibrillation and National Institute of Health Stroke Scale. (C) Hazard ratio for first-ever ischemic stroke mortality; (D) Hazard ratio for first-ever hemorrhagic stroke mortality. CKD: chronic kidney disease.

Fig 2 showed the Adjusted Kaplan-Meier survival curves for first-ever IS and first-ever HS according to different CKD stages. CKD G5 patients had the worst survival outcomes among both IS and HS patients.

To clarify whether CKD stage is a strong predictor of post-stroke mortality, the mortality HR of each CKD stage was calculated using the Cox proportional hazard model. We adjusted for NIHSS score, age, sex, hypertension, DM, atrial fibrillation, and malignancies in both IS and HS patients.

In the IS group, the mortality rate increased with CKD progression. Compared to G1 patients, CKD G3, G4, and G5 patients all had significantly higher mortality risks. The one-month mortality HR was 2.36 (1.27–4.39; 95% CI), and the one-year mortality HR was 5.28 (3.94–7.08; 95% CI) for CKD G5 patients (Table 2). Fig 2 shows the forest plots of adjusted HRs for mortality after first-ever IS.

**Table 2:** Hazard ratios for first-ever stroke mortality according to different CKD stages.

**Ischemic stroke**	**1-month mortality**			**1-year mortality**		
	HR	95% CI	*p*-value	HR	95% CI	*p*-value
G1	1			1		
G2	1.16	0.76–1.77	0.5003	1.17	0.95–1.44	0.134
CKD G3	1.70	1.10–2.64	0.0170*	1.89	1.53–2.35	<.0001*
CKD G4	1.96	1.05–3.64	0.0338*	2.72	2.00–3.71	<.0001*
CKD G5	2.36	1.27–4.39	0.0067*	5.28	3.94–7.08	<.0001*
**Hemorrhagic stroke**	**1-month mortality**			**1-year mortality**		
	HR	95% CI	*p*-value	HR	95% CI	*p*-value
G1	1			1		
G2	1.10	0.73–1.67	0.6472	1.18	0.85–1.63	0.3262
CKD G3	1.34	0.85–2.13	0.2119	1.61	1.12–2.31	0.0105*
CKD G4	1.06	0.42–2.70	0.8964	1.72	0.86–3.45	0.1274
CKD G5	2.17	1.33–3.54	0.0019*	3.03	2.03–4.54	<.0001*

Adjusted for age, sex, hypertension, diabetes mellitus, atrial fibrillation, malignancies, and National Institute of Health Stroke Scale.

*p<0.0.5

CKD: chronic kidney disease; HR: hazard ratio; CI: confidence interval

In the HS group, CKD G5 patients also had the highest HR of mortality compared to patients with other CKD stages. Compared to G1, the one-month mortality HR for the CKD G5 patients was 2.17 (1.33–3.54; 95% CI), and the one-year mortality HR was 3.03 (2.03–4.54; 95% CI) (Table 2). The adjusted HRs for mortality after first-ever HS according to different CKD stages are shown in Fig 2.

### Causes of death according to CKD stages in first-ever ischemic stroke patients

**Fig 3:**
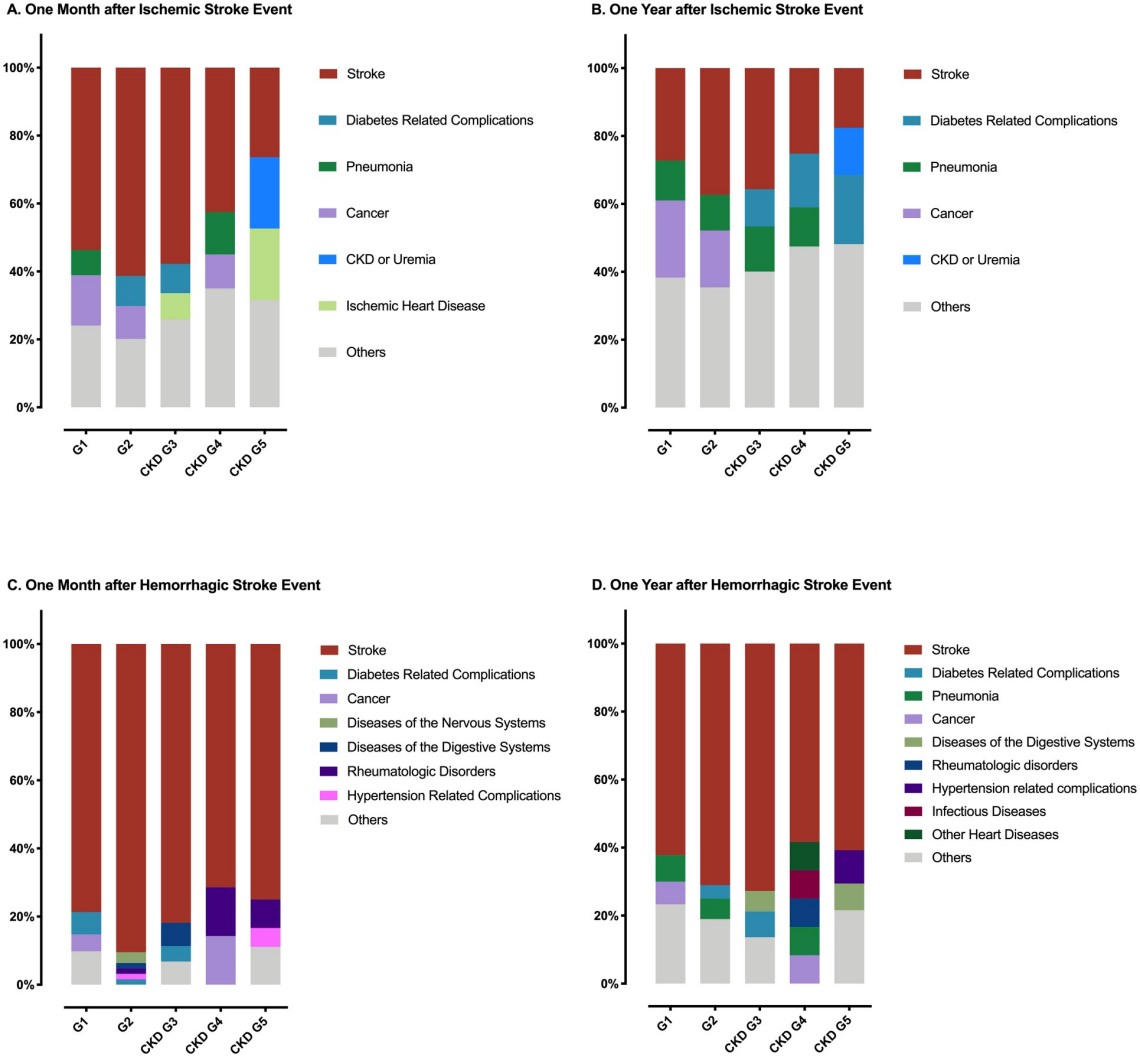
The proportion of different causes of death after the first-ever stroke. Leading causes of death after first-ever stroke. (A) Within one month after the first-ever ischemic stroke event; (B) within one year after the first-ever ischemic stroke event; (C) within one month after the first-ever hemorrhagic stroke event; (D) within one year after the first-ever hemorrhagic stroke event. CKD: chronic kidney disease.

By linking to the National Registry of Deaths Database of Taiwan, major causes of death in our study cohort were further analyzed. Table 3 shows the numbers and proportions of the leading causes of death in patients with different CKD stages after first-ever IS, and Fig 3 shows the unadjusted relative percentages of causes of death according to different CKD stages.

**Table 3:** Top 3 causes of mortality in first-ever stroke patients with different CKD stages.

Ischemic stroke	G1		G2		CKD G3		CKD G4		CKD G5	
1–30 day mortality	Disease	n (%)	Disease	n (%)	Disease	n (%)	Disease	n (%)	Disease	n (%)
	CVA	18 (50.00)	CVA	48 (63.16)	CVA	44 (56.41)	CVA	9 (42.86)	CVA	5 (26.32)
	Cancer	5 (13.89)	Cancer	9 (11.84)	DM	7 (8.97)	Cancer	4 (19.05)	CKD	4 (21.05)
	Pneumonia	4 (11.11)	Other heart diseases	7 (9.21)	Other heart diseases	7 (8.97)	Pneumonia	2 (9.52)	IHD	4 (21.05)
	Total mortality	36	Total mortality	76	Total mortality	78	Total mortality	21	Total mortality	19
1–365 day mortality	CVA	40 (27.59)	CVA	102 (34.23)	CVA	100 (34.01)	CVA	18 (25.71)	DM	18 (23.08)
	Cancer	36 (24.83)	Cancer	62 (20.81)	Pneumonia	42 (14.29)	DM	12 (17.14)	CVA	13 (16.67)
	Pneumonia	19 (13.1)	Pneumonia	29 (9.73)	Cancer	34 (11.56)	Cancer	11 (15.71)	CKD	10 (12.82)
	Total mortality	145	Total mortality	298	Total mortality	294	Total mortality	70	Total mortality	78
Hemorrhagic stroke	CKD G1		CKD G2		CKD G3		CKD G4		CKD G5	
1–30 day mortality	Disease	n(%)	Disease	n(%)	Disease	n(%)	Disease	n(%)	Disease	n(%)
	CVA	48 (78.69)	CVA	57 (90.48)	CVA	36 (81.82)	CVA	5 (71.43)	CVA	27 (75.00)
	DM	4 (6.56)	Nervous systems	2 (3.17)	GI	3 (6.82)	Cancer	1 (14.29)	Rheuma	3 (8.33)
	Cancer	3 (4.92)	DM, GI, HTN	4 (6.35)	DM	2 (4.55)	Rheuma	1 (14.29)	HTN	2 (5.56)
	Total mortality	61	Total mortality	63	Total mortality	44	Total mortality	7	Total mortality	36
1–365 day mortality	CVA	56 (62.22)	CVA	71 (71)	CVA	48 (72.73)	CVA	7 (58.33)	CVA	31 (60.78)
	Pneumonia	7 (7.78)	Pneumonia	6 (6)	DM	5 (7.58)	Pneumonia	1 (8.3)	HTN	5 (9.8)
	Cancer	6 (6.67)	DM	4 (4)	GI	4 (6.06)	Infection Other heart diseases, Cancer Rheuma	1 (8.3) 1 (8.3) 1 (8.3) 1 (8.3)	GI	4 (7.84)
	Total mortality	90	Total mortality	100	Total mortality	66	Total mortality	12	Total mortality	51

CVA: cerebrovascular accident; DM: diabetes mellitus and related complications; CKD: chronic kidney disease-related complications; IHD: ischemic heart disease; GI: digestive system diseases and related complications; IHD: ischemic heart disease; HTN: hypertension and related complications; Rheuma: rheumatological disorders

In one-month mortality, stroke itself was the most common cause of death after first-ever IS in all CKD groups. Stroke accounted for more than half of deaths in normal or early CKD (G1 to CKD G3) patients (50%, 63.16%, 56.41% in G1, G2, CKD G3, respectively), but in CKD G5, the proportion of deaths from stroke decreased to less than 27%. CKD-related complications and ischemic heart disease (IHD) explained 21% of deaths in CKD G5 patients, respectively.

In one-year mortality, stroke remained the most common cause of death in G1 to CKD G4 patients, but not in CKD G5 patients. Instead, DM-related complications became the most common (23.08%) cause of death in CKD G5 patients, and CKD-related mortality raised to the third place (12.82%) in this group. The other major causes of death in early CKD patients included cancer (11.56–24.83% of deaths) and pneumonia (9.73–14.29% of deaths). In both one-month and one-year mortality, there was an inverse association between eGFR decrement and the proportion of deaths from stroke among patients with G2 to CKD G5.

### Causes of death according to CKD stages in first-ever hemorrhagic stroke patients

Among these CKD patients, the primary cause of death after first-ever HS was stroke itself (Table 3 and Fig 3). Stroke accounted for more than half of all deaths in one-month (71.43–90.48%) and one-year (58.33–72.73%) post-stroke mortality in our patients. The proportion of deaths caused by stroke showed no significant difference among these CKD stages.

## Discussion

In this large population-based cohort study, we found the leading causes of death after IS varied according to different CKD stages. A thorough search of the literature yielded no study regarding this issue. Our study showed stroke was the top leading cause of post-stroke death in any stage of CKD at one month and one year in both ischemic and hemorrhagic stroke, suggesting the importance of secondary stroke prevention in this population. Less proportion of patients died due to stroke itself in our CKD G5 patients than early-stage CKD patients after IS. In the year after an IS event, DM and CKD became the leading causes of death in our late-stage CKD patients. Clinicians may need to imply different treatment strategies according to stroke patient’s CKD severity.

Cancer was one of the leading causes of death within one year of IS event in this study. More and more evidence show these two disease entities are related. One study reported three-month cumulative incidence of stroke was 5% in patients with lung cancer compared to 1.2% in controls [17]. Another study discovered that cancer incidence was higher among ischemic stroke patients compared with general population [18]. Besides, cancer patients have more fetal stroke than normal population [19]. Cryptogenic stroke patients may benefit from screening for undiscovered cancer to improve their survival.

Previous study confirmed that IS was independently associated with increased risk of incident major cardiovascular event [20]. Our data also showed that IHD is one of the major causes of death within one month after IS in our CKD G5 patients, but not the top causes of death of one-year mortality. Fatal IHD was an unneglectable condition during acute stage of an IS event. CKD, IHD, and IS all share lots of common risk factors. Epidemiological studies have shown that cardiovascular disease is the most common cause of death in CKD population and declining of eGFR is associated with higher risk of cardiovascular death [21, 22]. These might explain why more CKD G5 patients died due to IHD within one month after their first-ever IS in our study. Pneumonia was not one of the main causes of death in general CKD patients in earlier studies [21], but our data revealed that up to 14% of one-year deaths were from pneumonia in early CKD patients after IS. As a serious complication after stroke, pneumonia may increase the in-hospital mortality rate [23], especially in those with greater disease severity [24]. Swallowing dysfunction and physiological dependence after stroke may increase risk for aspiration pneumonia [25]. Compromised immunity in CKD patients can also contribute to the high prevalence of pneumonia. Early screening of dysphagia and prompt diagnosis of infectious disease is crucial for patients with CKD after stroke in both acute and chronic phases [26].

Our study also showed that patients with late-stage CKD had worse post-stroke outcomes with higher NIHSS scores and greater post-stroke disability. This result is similar to several previous studies [9, 11]. The mechanisms underlying poor stroke outcome in CKD patients might be multifactorial, including the tendency of hemorrhagic transformation due to high bleeding risk [27] and more severe vascular damage due to increased inflammatory status and endothelial cell dysfunction [28]. Like other study [12], our data also showed increased mortality rates along with CKD progression after first-ever stroke. The adjusted HRs of post-stroke mortality were significantly higher in later CKD stages, and this phenomenon was more prominent in IS than in HS patients. CKD severity is a strong independent predictor of both one-month and one-year mortality after IS. Previous study showed NIHSS is a good predictor for HS outcome [29]. After adjusting NIHSS and other risk factors, our data showed that CKD G5 had negative impact on both one-month and one-year post HS mortality. With some similarities to other studies, there were some advantages to our study design. First, we enrolled both IS and HS patients and analyzed their clinical outcomes and mortality rates separately. From our data, the severity of CKD had more impact on mortality in IS than in HS. Second, we used the CKD-EPI equation for eGFR calculation, which has been shown to be more accurate for risk prediction than the MDRD equation [30, 31]. Third, this was a multicenter study and enrolled large numbers of patients from both urban and rural areas with minimal selection bias.

Current study showed a higher mortality rate in first-ever HS patients than in IS patients, and most of the deaths occurred in the first month after HS. These findings are consistent with other studies [32]. The major causes of death after HS according to CKD stages did not show much heterogeneity as IS. Stroke itself accounted for more than 70% of deaths during the first month and persisted as the top cause of death up to one year after HS across all the CKD stages. Hemorrhagic stroke *per se* remained the most crucial factor for the prognosis. It is worth noting that late-stage CKD patients have higher mortality rates and may need specific care.

There were some limitations of this study. First, the National Death Registry in Taiwan used an underlying-cause-of-death method and was coded in ICD-10-CM, which was developed by the World Health Organization. One report stated that this method might underestimate stroke as the cause of death [33], and miscoding might also exist in the registry [34]. Second, this study was conducted in Taiwan, and 95% of the population is Han-Chinese. The external generalizability of our result may be limited, especially generalization to Caucasian and African populations. Third, this was a database study, some detailed clinical data was not included in our database, we did not have urine albumin to creatinine ratio nor kidney imaging data. We were unable to distinguish CKD or non-CKD in our G1 and G2 patients.

## Conclusion

Late-stage CKD patients had worse symptoms, poorer outcomes with more functional dependence, and higher mortality rates after first-ever stoke. Differences in major causes of death after IS existed between early- and late-stage CKD patients. Cerebrovascular disease was the top cause of death in most CKD patients who experienced a stroke, but the proportion of stroke-related deaths declined with CKD progression. The leading cause of death after HS was stroke itself across all CKD stage patients. Patients with low eGFR need more intensive and comprehensive care to improve their clinical outcomes. Knowing the causes of death after stroke in CKD patients may help clinicians to imply different treatment strategies to decrease their high mortality.

## Supporting information

S1 Table ICD-10-CM codes for underlying causes of death. (DOCX)

S2 Table Stroke subtypes of CKD stages in first-ever ischemic stroke. (DOCX)

S3 Table Post hoc analysis of clinical severity among different CKD stages after first-ever ischemic stroke. (DOCX)

S4 Table Post hoc analysis of clinical severity among different CKD stages after first-ever hemorrhagic stroke. (DOCX)
