# Supplementary material for: Impact of chronic kidney disease severity on causes of death after first-ever stroke: A population-based study using nationwide data linkage
Source: PLoS One. 2020 Nov 19;15(11):e0241891. doi: 10.1371/journal.pone.0241891 (PMC7676709; doi:10.1371/journal.pone.0241891)
Supplement: S1 Table — (DOCX) [file pone.0241891.s001.docx]

**S1 Table. ICD-10-CM codes for underlying causes of death**

| **Diseases** | **ICD10-CM Code** |
| --- | --- |
| Intestinal infectious diseases | A00-A09 |
| Tuberculosis | A15-A19 |
| Septicemia | A40-A41 |
| Viral hepatitis | B15-B19 |
| Human immunodeficiency virus disease | B20-B24 |
| Cancer | C00-C97 |
| In situ neoplasms and benign neoplasms | D00-D48 |
| Anemia | D50-D64 |
| Diabetes mellitus | E10-E14 |
| Vascular dementia and unspecified dementia | F01-F03 |
| Meningitis | G00, G03 |
| Spinal muscular atrophy | G12 |
| Parkinson’s disease | G20-G21 |
| Alzheimer’s disease | G30 |
| Hypertension | I10-I15 |
| Ischemic heart disease | I20-I25 |
| Pulmonary hypertension | I27 |
| Other forms of heart disease | I30-I52 |
| cerebrovascular accident | I60-I69 |
| Atherosclerosis | I70 |
| Aortic aneurysm and dissection | I71 |
| Influenza | J10-J11 |
| Pneumonia | J12-J18 |
| Acute bronchitis and bronchiolitis | J20-J21 |
| Chronic lower respiratory diseases | J40-J47 |
| Lung diseases due to external agents | J60-J69 |
| Diseases of the digestive system | K00-K99 |
| Diseases of the skin and subcutaneous tissue | L00-L99 |
| Rheumatologic disorders | M00-M99 |
| Kidney diseases | N00-N07, N17-N19, N25-N27 |
| Pregnancy, childbirth, puerperium, and related complications | O00-O99 |
| Certain conditions originating in the perinatal period | P00-P96 |
| Age-related physical debility | Q00-Q99 |
| **Diseases** | **ICD10-CM code** |
| Age-related physical debility | R54 |
| Sudden infant death syndrome | R95 |
| Accident | V01-X59, Y85-Y86 |
| Suicide | X60-X84, Y87.0 |
| Assault | X85-Y09, Y87.1 |
