# Supplementary material for: Impact of chronic kidney disease severity on causes of death after first-ever stroke: A population-based study using nationwide data linkage
Source: PLoS One. 2020 Nov 19;15(11):e0241891. doi: 10.1371/journal.pone.0241891 (PMC7676709; doi:10.1371/journal.pone.0241891)
Supplement: S2 Table — (DOCX) [file pone.0241891.s002.docx]

**S2 Table. Stroke Subtypes of CKD stages in first-ever ischemic stroke.**

|  | Total | G1 | G2 | CKD G3 | CKD G4 | CKD G5 | p value |
| --- | --- | --- | --- | --- | --- | --- | --- |
| eGFR (ml/min/1.73m^2^) |  | ≥90 | 60-89 | 30-59 | 15-29 | <15 |  |
| Number | 9,878 | 3,372 | 3,957 | 1,943 | 309 | 297 |  |
| Stroke subtype |  |  |  |  |  |  | <.0001 |
| CE | 1310(13.3) | 307(9.1) | 535(13.5) | 350(18.0) | 67(21.7) | 51(17.2) |  |
| CE+LAA | 379(3.8) | 69(2.0) | 170(4.3) | 112(5.8) | 20(6.5) | 8(2.7) |  |
| LAA | 1952(19.8) | 681(20.2) | 737(18.6) | 437(22.5) | 50(16.2) | 47(15.8) |  |
| SVO | 2712(27.5) | 962(28.5) | 1121(28.3) | 470(24.2) | 70(22.7) | 89(30.0) |  |
| TIA | 446(4.5) | 175(5.2) | 174(4.4) | 66(3.4) | 18(5.8) | 13(4.4) |  |
| UDE | 3079(31.2) | 1178(34.9) | 1220(30.8) | 508(26.1) | 84(27.2) | 89(30.0) |  |

CE: cardiac embolism; LAA: large artery atherosclerosis ; SVO: small vessel occlusion ; TIA: transient ischemic attack; UDE: undetermined etiology
