# Supplementary material for: Impact of chronic kidney disease severity on causes of death after first-ever stroke: A population-based study using nationwide data linkage
Source: PLoS One. 2020 Nov 19;15(11):e0241891. doi: 10.1371/journal.pone.0241891 (PMC7676709; doi:10.1371/journal.pone.0241891)
Supplement: S3 Table — (DOCX) [file pone.0241891.s003.docx]

**S3 Table. Post hoc analysis of clinical severity among different CKD stages after first-ever ischemic stroke**

| **Post hoc analysis** |  | ***p* value** |
| --- | --- | --- |
| Admission NIHSS | G1 versus G2 | 0.0944 |
|  | G1 versus CKD G3 | <.0001 |
|  | G1 versus CKD G4 | <.0001 |
|  | G1 versus CKD G5 | <.0001 |
|  | G2 versus CKD G3 | <.0001 |
|  | G2 versus CKD G4 | <.0001 |
|  | G2 versus CKD G5 | <.0001 |
| Discharge BI | G1 versus G2 | 0.0014 |
|  | G1 versus CKD G3 | <.0001 |
|  | G1 versus CKD G4 | <.0001 |
|  | G1 versus CKD G5 | <.0001 |
|  | G2 versus CKD G3 | <.0001 |
|  | G2 versus CKD G4 | <.0001 |
|  | G2 versus CKD G5 | <.0001 |
| Discharge MRS | G1 versus G2 | <.0001 |
|  | G1 versus CKD G3 | <.0001 |
|  | G1 versus CKD G4 | <.0001 |
|  | G1 versus CKD G5 | <.0001 |
|  | G2 versus CKD G3 | <.0001 |
|  | G2 versus CKD G4 | <.0001 |
|  | G2 versus CKD G5 | <.0001 |

NIHSS: National Institute of Health Stroke Scale; BI: Barthal index; mRS: modified Ranking Scale
