# Supplementary material for: Impact of chronic kidney disease severity on causes of death after first-ever stroke: A population-based study using nationwide data linkage
Source: PLoS One. 2020 Nov 19;15(11):e0241891. doi: 10.1371/journal.pone.0241891 (PMC7676709; doi:10.1371/journal.pone.0241891)
Supplement: S4 Table — (DOCX) [file pone.0241891.s004.docx]

**S4 Table. Post hoc analysis of clinical severity among different CKD stages after first-ever hemorrhagic stroke**

| **Post hoc analysis** |  | ***p value*** |
| --- | --- | --- |
| Admission NIHSS | G1 versus G2 | 0.9550 |
|  | G1 versus CKD G3 | <0.0210 |
|  | G1 versus CKD G4 | <0.4612 |
|  | G1 versus CKD G5 | <.0001 |
|  | G2 versus CKD G3 | 0.1033 |
|  | G2 versus CKD G4 | 0.6446 |
|  | G2 versus CKD G5 | <.0001 |
| Discharge BI | G1 versus G2 | 0.9976 |
|  | G1 versus CKD G3 | <0.0173 |
|  | G1 versus CKD G4 | <0.3725 |
|  | G1 versus CKD G5 | <.0001 |
|  | G2 versus CKD G3 | 0.0420 |
|  | G2 versus CKD G4 | 0.4545 |
|  | G2 versus CKD G5 | <.0001 |
| Discharge MRS | G1 versus G2 | 1.0000 |
|  | G1 versus CKD G3 | 0.0043 |
|  | G1 versus CKD G4 | <0.3695 |
|  | G1 versus CKD G5 | <.0001 |
|  | G2 versus CKD G3 | 0.0054 |
|  | G2 versus CKD G4 | 0.3747 |
|  | G2 versus CKD G5 | <.0001 |
